# Plan-Do-Check-Action Circulation Combined with Accelerated Rehabilitation Nursing under Computed Tomography in Prevention and Control of Hospital Infection in Elderly Patients Undergoing Elective Orthopedic Surgery

**DOI:** 10.1155/2022/4574730

**Published:** 2022-04-25

**Authors:** Liguo Zhao, Lianghong Hu, Zhangxian Li, Fenyan Deng

**Affiliations:** ^1^Fever Clinic, The Third Affiliated Hospital Hengyang Medical School, University of South China, Hengyang 421900, Hunan, China; ^2^Department of Nursing, The Third Affiliated Hospital Hengyang Medical School, University of South China, Hengyang 421900, Hunan, China; ^3^Department of Spinal Surgery, The Third Affiliated Hospital Hengyang Medical School, University of South China, Hengyang 421900, Hunan, China

## Abstract

To explore the adoption of plan-do-check-action (PDCA) circulation combined with accelerated rehabilitation nursing based on gemstone spectral imaging computed tomography (GSICT) in the prevention and control of hospital infection in the elderly patients undergoing the elective orthopedic surgery, 80 elderly patients who underwent the elective orthopedic surgery in the hospital were selected. Then, according to the randomized controlled principle, these 80 patients were divided into control group (40 cases) with conventional nursing and observation group (40 cases) with accelerated rehabilitation surgical nursing combined with PDCA circulation. All the patients underwent the GSICT examination without any contraindicators. Compared with the conventional CT scan, metal artifacts in GSICT were considerably reduced. In the images processed by GSI and metal artifacts reduction system (MARS), metal artifacts were basically eliminated and the positions, forms, and edges of metal artifacts in the human body were clearly presented. Hospital infection occurred in 1 (2.5%) patient in the observation group and 5 (12.5%) patients in the control group, and the difference was statistically significant (*P* < 0.05). In terms of temperature increase, patients in control group (37.5%) had a remarkably higher value than that of observation group (7.5%). The increase rate of white blood cell (WBC) count in control group (12.5%) was obviously higher than that in observation group (2.5%). Besides, the differences were statistically significant (*P* < 0.05). After PDCA circulation combined with accelerated rehabilitation nursing mode was applied, the hospitalization time of observation group (5.3 ± 2.4 days) was markedly lower than that of control group (9.7 ± 3.8 days). Moreover, the total hospitalization cost of observation group (791.44 yuan) was notably lower than that of control group (4068.96 yuan), with significant differences (*P* < 0.05). Nursing satisfaction in observation group (92.5%) was higher than that in control group (77.5%), and the difference was statistically significant (*P* < 0.05). In short, GSICT could effectively reduce beam hardening artifacts and metal implant artifacts and improve image quality. Furthermore, accelerated rehabilitation nursing combined with PDCA circulation could effectively reduce the incidence of hospital infection and improve nursing satisfaction.

## 1. Introduction

Hospital infection refers to infections acquired by patients in hospital, including the infections acquired during hospitalization and the infections acquired in hospital and developed after discharge [1]. Patients who undergo the orthopedic surgery suffer from hunger, anesthesia, pain, and many other stimuli. Besides, the incidence of postoperative hospital infection is high, which seriously affects the rehabilitation process of patients [2]. The concept of surgical site infection (SSI) was first proposed by the U.S. Center of Disease Control in the late 20^th^ century. According to the data, the incidence of SSI ranked third in hospital infection, which accounts for about 15% of all hospital infection patients [3]. According to the domestic and international statistical data, during surgery, the overall incidence of SSI in patients is 2%–20%, and the incidence of SSI in clean surgery is the lowest [4–6]. Conventional perioperative orthopedic nursing is far from meeting patients' rehabilitation needs, and it is imperative to seek a new, rapid, and scientific nursing mode. The function of accelerated rehabilitation nursing in orthopedics is that it can effectively promote the rehabilitation of patients and reduce the incidence of hospital sense after orthopedics surgery [7–9]. In the process of clinical nursing, it is difficult to implement each step of accelerated rehabilitation surgery in nursing management work.

Accelerated rehabilitation nursing refers to a concept of accelerated physical and psychological rehabilitation in the perioperative period, based on a series of evidence of medicine and combined with multiple disciplines, on the premise of ensuring safety, reducing surgical stress response, hospitalization time, and cost [10]. Through systematic meta-analysis, Rao et al. [11] concluded that compared with conventional nursing, accelerated rehabilitation nursing could effectively reduce the occurrence of complications such as deep vein thrombosis and infection. Additionally, Shao et al. [12] found that accelerated rehabilitation nursing could effectively reduce the surgical trauma of patients who underwent the laparoscopic radical resection of rectal cancer and reduce the surgical inflammatory stress response, thus promoting the postoperative rehabilitation. At present, the main reason for accelerated rehabilitation nursing is providing intervention measures for various risk factors, and effectively reducing the hospitalization time and the risk of iatrogenic infection.

The plan-do-check-action (PDCA) circulation method is a quality management program proposed by Deming, an American management scientist. Through plan, do, check, and action, the implementation of the whole nursing step is mainly preformed in a circulation and improved continuously. According to effective research results, the potential nursing problems in the nursing work are found out. Then, the corresponding nursing interventions are given to address the existing nursing problems, so as to improve the risk awareness of nursing staffs, and effectively reduce the occurrence of adverse nursing events [13–15]. Xu et al. [16] found that due to the adoption of PDCA circulation management in the management of hospital infection in orthopedic surgery, the hospital infection rate and the surgical infection rate of patients were reduced, and the awareness rate of hospital infection and qualified rate of hand hygiene of medical staff were improved as well as the satisfaction rate of patients. Moreover, the adoption of PDCA circulation to implement cluster interventions was helpful to improve hand hygiene compliance effectively, thus reducing the incidence of hospital infections, which was explored by Demirel [17].

When patients who have undergone the elective orthopedic surgery receive the postoperative computed tomography (CT), radially diffused starlike and stepped artifacts are often found, due to the use of metal devices during internal fixation, such as intramedullary steel nails, metal replacement joints, and internal fixation plates. The great difficulties to the reconstruction of CT images are brought by the generation of artifacts, which causes misdiagnosis [18]. Gemstone spectral imaging computed tomography (GSICT) can solve the problem effectively. The dual energy process in the projection data space is completed by this technique. The metal artifacts are removed by the metal artifact elimination reconstruction technique. Furthermore, GSICT is widely used in postoperative examination of patients who have undergone the elective orthopedic surgery [19, 20].

The incidence of SSI after elective orthopedic surgery is influenced by many factors. Different surgical methods are varied with the incidence of SSI, but these factors can be prevented. Nurses are the ones who accompany orthopedic patients for the longest time during hospitalization. Therefore, according to these factors, certain preventive measures are implemented to avoid the occurrence of hospital infections. At present, there is no adoption of accelerated rehabilitation nursing under CT examination combined with PDCA circulation quality improvement in the prevention and control of postoperative hospital infections in elderly patients undergoing the elective orthopedic surgery. Hence, it was necessary to implement this adoption. Additionally, it could also provide a more safe, scientific, and effective accelerated rehabilitation nursing mode for the prevention and control of postoperative hospital infections in elderly patients who underwent elective orthopedic surgery. The level of prevention and control of orthopedic hospital infection in this region is improved, the medical resources are saved, and the burden of disease caused by hospital infection in orthopedic wards is also reduced. Therefore, accelerated rehabilitation nursing under CT examination combined with PDCA circulation is widely applied in the prospect.

## 2. Methods

### 2.1. Subjects of the Study

Eighty elderly patients who were admitted to the hospital from January 2019 to January 2021 for elective orthopedic surgery were selected. According to the randomized controlled principle, these 80 patients were divided into control group (40 cases) with conventional nursing and observation group (40 cases) with accelerated rehabilitation surgical nursing combined with PDCA circulation. There were 43 male patients and 37 female patients, and the mean age was 65.32 ± 9.74 years old. There is no difference in sex and age between the two groups, which is comparable. This study had been approved by the ethics committee of hospital. All the patients and their families knew about this study and signed the informed consent form.

The inclusion criteria were as follows: (i) patients older than 45 years old; (ii) patients scheduled for elective orthopedic surgery; (iii) patients in good physical condition and could tolerate surgery; and (iv) patients who informed about the study and agreed to participate in the study.0

The exclusion criteria were as follows: (i) those with poor compliance and (ii) those diagnosed with other organic diseases, such as mental disorders and liver and kidney dysfunction.

### 2.2. CT Examination

Patients were scanned by a high-definition energy spectrum CT, and the gemstone spectral imaging (GSI) mode was selected. The scan parameters were as follows. The tube current was 330 mA, the tube voltage was 120 kV, the scanning field was 25 cm, the tube speed was 350 ms, the spacing and layer thickness were 5 mm, the width of the single detector after reconstruction was 0.5 mm, and the pitch was 0.95 : 1. Besides, metal artifacts were removed by the metal-artifacts reduction system (MARS) technology. The window position was fixed at 420Hu and the window width was 2200Hu during the scanning process.

### 2.3. Specific Methods

In control group, patients were treated with conventional nursing, and they were informed to quit smoking and drinking before surgery, supple nutrition, and pay attention to postoperative wound care. Patients in observation group were treated with the improved PDCA circulation based on accelerated rehabilitation surgical nursing.

The specific contents of accelerated rehabilitation surgical nursing were as follows. Before the surgery: (i) Medical history was inquired in detail, and the recent history of respiratory tract infection (RTI) and periodontitis infection was also asked. (ii) Nutritional support was enhanced. Through the diet of high protein and high vitamin, patients' hypoproteinemia and anemia were corrected and their resistance was improved. (iii) Health education was emphasized. Two weeks before surgery, patients were told to quit smoking and they did cough exercises to improve lung function under the instrument. (iv) After admission, the skin of the surgical site was maintained clean every day. The prophylactic use of antibiotics was followed by doctor's advice before surgery, and intravenous infusion was started 30–60 minutes before surgery.

After the surgery: (i) Patients' anemia and hypoproteinemia were corrected continuously and promptly. The diet was the same as that before the surgery. (ii) Blood glucose of patients with diabetes was continuously monitored after surgery. Surgical stress could cause diabetic patients to develop insulin resistance and remain hyperglycemic for weeks. The risk of incision-related complications was increased. (iii) Incision management was strengthened. After surgery, it should strengthen muscle contraction exercise, promote venous and lymphatic reflux, and reduce edema of affected limbs. (iv) The management of hospital discharge. After discharge, patients were followed up closely 2-3 weeks, 1 month, 3 months, 6 months, 1 year after surgery, and every year thereafter, patients needed to come to the outpatient clinic for re-examination to check the surgical site for abnormal pain, redness, and skin temperature rise, and they were guided to do functional exercise.

PDCA circulation was classified into four stages: plan, implementation, inspection, and treatment. In the accelerated rehabilitation nursing of elderly patients who underwent elective orthopedic surgery, nursing was classified into four stages by the nurses in view of the existing problems. The first one was the planning stage, which included present situation analysis, cause investigation, finding the influencing factors, and correction and prevention. The second one was the implementation stage to implement the plan. The third one was the inspection stage to investigate the results. The last one was the treatment stage to summarize experience and set standards. This circulation was repeated to optimize the rehabilitation surgical nursing process after elective surgery and reduce the occurrence of orthopedic hospital infection.

### 2.4. Observation Indicators

Incision infection rate, body temperature elevation rate (body temperature elevation above 38°C) at three days after surgery, and white blood cell (WBC) elevation rate (WBC > 10.0 × 10^9^) were compared between the two groups. The length of hospital stay, total hospital cost, and nursing satisfaction were also compared between the two groups. Nursing satisfaction was evaluated by a self-made questionnaire of the department, with 0–100 points, which included satisfied score (>80 points), basically satisfied score (65–80 points), and dissatisfied score (<65 points). Total satisfaction = (satisfied cases + basic satisfied cases)/40 × 100%.

### 2.5. Image Processing and Observation Comparison

The conventional CT scan was not implemented repeatedly. Patients' pervious conventional CT scan data were transferred from hospital information system (HIS) online back to AW 4.5 post-processing workstation for a variety of recombination treatments, to reduce the amount of radiation to the patients including volume rendering (VR), maximum intensity projection (MIP), and multiplanar reformation (MPR). The obtained images were saved for comparison with those obtained from GSICT. The feasibility processing was implemented on the GSI scan image data transmitted to AW 4.5 post-processing workstation. The GSI viewer processing software was used for analysis, and the single energy image suitable for keV adjustment was selected. Then, images from the same layers as shoes from a conventional CT scan were found and compared. Furthermore, a different software was selected to process and observe the different cases, especially for careful reading and study of axial images, and to select the best way to evaluate the scanned images.

### 2.6. Statistical Methods

SPSS 22.0 was employed for data statistics and analysis. The measurement data $\left(\bar{x}\pm s\right)$ were compared by the independent sample *t*-test. The enumeration data (*n*, %) were tested by *χ*^2^ test. When *P* < 0.05, it meant that the difference was statistically significant.

## 3. Results

### 3.1. Results of the CT Scan

**Figure 1:**
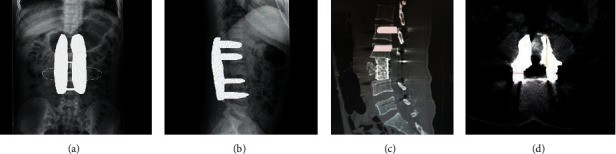
Conventional CT scan images. (a) Coronal position of lumbar vertebra. (b) Sagittal position of lumbar vertebra. (c) Sagittal position of thoracic vertebra. (d) Transverse position of lumbar vertebra. Many metal artifacts were observed, and the relationship among artificial joints and the position of internal fixation nails were blurred.

Through a variety of post-processing software, the metal artifacts caused by the presence of metal products in the body such as internal fixation nails and artificial joints could not be eliminated by the conventional CT scan. The accuracy of postoperative examination and diagnosis of orthopedic patients were reduced by the unclear relationship among artificial joints and the position of internal fixation nails, and even misdiagnosis was caused. The great difficulties were brought for orthopedic surgeons to follow-up patients after surgery (Figure 1).

**Figure 2:**
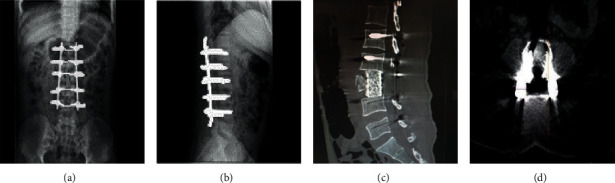
GSICT  scan images processed by GSI and MARS. (a) Coronal position of lumbar vertebra. (b) Sagittal position of lumbar vertebra. (c) Sagittal position of thoracic vertebra. (d) Transverse position of lumbar vertebra. Compared with conventional CT  scan images, the image clarity of the above four figures was obviously improved, and metal radial artifacts were basically eliminated. The relationship among artificial joints and the position of internal fixation nails were shown to facilitate observation.

Compared with the conventional CT scan, metal artifacts in GSICT were considerably reduced. In the images processed by GSI and MARS, metal artifacts were basically eliminated, and the positions, forms, and edges of metal artifacts in human body were clearly presented. GSICT helped to evaluate the effects of fracture reduction and internal fixation and improve the accuracy of diagnosis. Besides, it also helped to facilitate orthopedic surgeons to observe CT reexamination images and to evaluate the postoperative rehabilitation (Figure 2).

### 3.2. Results of Hospital Infection Incidence

**Figure 3:**
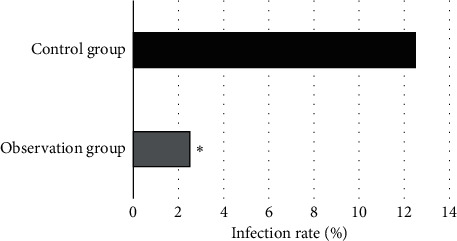
Incidence of hospital infection. ∗Compared with control group, *P* < 0.05.

In observation group, there was 1 patient (2.5%) who developed hospital infection, while in control group, there were 5 patients (12.5%) who developed hospital infection, including 1 patient with RTI, 2 patients with urinary tract infection (UTI), and 2 patients with incisional infection. Besides, the incidence of hospital infection in control group was evidently higher than that in observation group (*P* < 0.05) (Figure 3).

### 3.3. Rate of Elevated Body Temperature and Increased WBC Count

**Figure 4:**
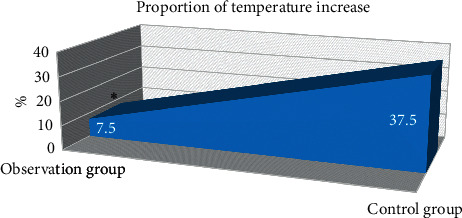
Proportion of temperature increase. ∗Compared with control group, *P* < 0.05.

In observation group, there were 3 patients (7.5%) with elevated body temperature (higher than 38°C), while there were 15 patients (37.5%) with elevated body temperature. Hence, in Figure 4, the rate of elevated body temperature was notably higher than that in observation group (*P* < 0.05).

**Figure 5:**
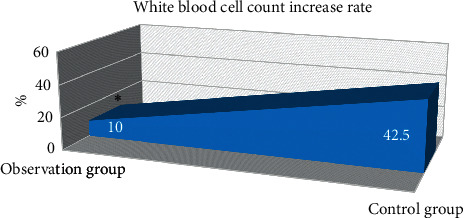
WBC count increase rate. ∗Compared with control group, *P* < 0.05.

Increased WBC was observed in 4 patients (2.5%) in the observation group (WBC > 10.0 × 10^9^), and in 17 patients (12.5%) in control group. Therefore, in Figure 5, the rate of increased WBC count in control group was remarkably higher than that in observation group (*P* < 0.05).

### 3.4. Length of Stay and Total Cost of Stay

**Figure 6:**
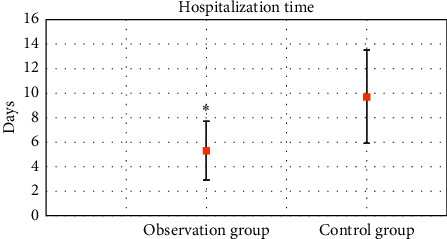
Comparison of hospitalization time. ∗Compared with control group, *P* < 0.05.

In Figure 6, after the adoption of PDCA circulation combined with accelerated rehabilitation nursing mode, the hospitalization time (5.3 ± 2.4 days) of the observation group was markedly lower than that of control group (9.7 ± 3.8 days) (*P* < 0.05).

In Table 1, the total hospitalization cost of observation group was 791.44 yuan, which was notably lower than that of control group (4,068.96 yuan) (*P* < 0.05).

### 3.5. Degree of Nursing Satisfaction

**Figure 7:**
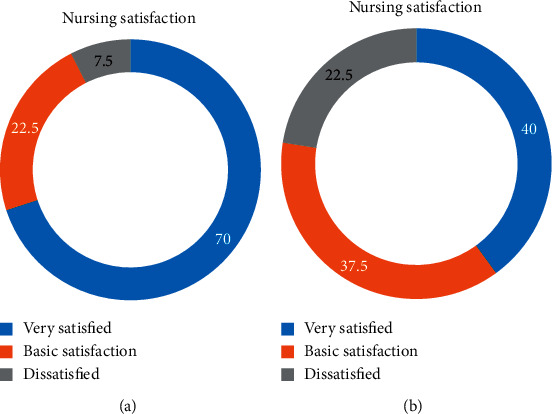
Nursing satisfaction. (a) Observation group. (b) Control group.

Table 2 and Figure 7 show that in observation group, 28 patients were very satisfied, 9 patients were basically satisfied, and 3 patients were dissatisfied, with a satisfaction rate of 92.5% (37/40). In control group, 16 patients were very satisfied, 15 patients were basically satisfied, and 9 patients were dissatisfied, with a satisfaction rate of 77.5% (31/40). Hence, the nursing satisfaction of observation group was higher than that of control group (*P* < 0.05).

## 4. Discussion

The imaging quality is determined by the detector which is the core part of CT. In the past 10 years, gemstones were used as detector materials for the GSICT developed by many scholars. The stability of gemstones is 20 times that of traditional rare Earth ceramic detectors and cadmium tungstate detectors. Gemstones have the characteristics of good air permeability and high purity, which ensure high quality of images and low dose of radiation. In the conventional CT scan, the diagnostic results are affected remarkably by artifacts caused by internal fixation plates and artificial joints [21]. Artifacts cause blurred imaging, which increases the difficulty of diagnosis. If diagnosis is wrong, premature exercise will cause the failure of elective orthopedic surgery. Additionally, delayed rehabilitation exercise will cause dyskinesia or muscle disuse atrophy [22]. Therefore, the removal of the artifacts becomes a vital issue to improve the image quality.

Compared with conventional CT equipment, HD energy spectrum CT imaging can realize single-photon imaging and material separation. HD energy spectrum CT can display fine lesions that cannot be found by conventional CT, which has been proved by several clinical cases. Meanwhile, density resolution has been improved. The sensitivity of small lesions is comparable to positron emission tomography (PET), and it has unique advantages in the specific diagnosis of lesions [23–25]. The HD energy spectrum CT scanning technology can effectively solve the problems of metal artifacts and improve the quality of CT images. Moreover, the technology requires that the CT scan is sampled at the same time to obtain double group projection data of objects. Hence, the conditions of high technology of hardware are necessary. MARS technology and single energy imaging technology are the main methods to remove artifacts by HD energy spectrum CT scanning technology. Furthermore, the MARS technology is designed to reduce image artifacts effectively by using software to control radiation through metal devices, generate low signals, and process the data simultaneously [26]. CT values of metal implants surrounding structures are precisely reflected by the single energy imaging technique combined with MARS. The CT value is proportional to the distance. The farther the distance is, the more it reflects the actual CT value of the tissue [27]. In the adoption of HD energy spectrum CT scanning technology, the region of 100–140 keV is the best region for GSI to eliminate metal implant artifacts. Additionally, this region is employed to improve the image quality obviously and to make the fine structures be imaged clearly.

PDCA circulation was born in the United States, based on the total quality management of the ideology and method. PDCA stands for plan, do, check, and action [28]. According to the adoption of PDCA circulation in the prevention and control of hospital infection in the surgical room, the various infection problems which have existed in the elderly patients are found out and solved in time, and the management quality of surgical room is improved because of the prevention and control function. Besides, the life safety of patients is also ensured. In the whole process of PDCA circulation nursing implementation, medical staff are required to have a high level of profession skills. The whole nursing structure is constantly optimized so that the quality of nursing work becomes more standardized and systematic. Moreover, the medical staff are also required to master the patients' disease development and postoperative recovery and to have an all-round communication with patients. With the deepening of the relationship of medical care, the quality of nursing work is improved. PDCA circulation combined with accelerated rehabilitation nursing was applied to patients who have undergone the elective orthopedic surgery, and the results were obtained as follows. The incision infection rate, body temperature, and WBC in observation group were lower than those in control group (*P* < 0.05). The length of hospitalization and total hospitalization cost in observation group were lower than those in control group (*P* < 0.05). The nursing satisfaction of observation group was higher than that of control group (*P* < 0.05). With the development of the PDCA circulation nursing model, the clinical manifestations of patients who underwent the elective orthopedic surgery were notably improved to shorten the hospital stay, which was investigated by Hua and Wang [29]. The results were consistent. According to PDCA circulation, the hospital stay was shortened, and the incidence of hospital infection was reduced. Furthermore, in the process of PDCA circulation nursing, problems were found and solved continually, and the implementations of every nursing detail were noticed. The executive ability of nursing was gradually improved while the incidence of hospital infection constantly decreased. The PDCA circulation nursing mode was applied in elective surgical treatment of orthopedics by Omar et al. [30]. The nursing quality was considerably improved, hospital stay was shortened, and the efficacy was improved. The results were also consistent.

Therefore, the adoption of PDCA circulation in the prevention and control of hospital infection in the surgical room is of high value, and it can be promoted and employed vigorously. Nevertheless, there are still some shortcomings. For instance, the sample size of the study was small; the HD energy spectrum CT scan required plenty of radiation energy, and the radiation dose received by patients was increased. Further studies and discussion are necessary in the future.

## 5. Conclusion

With the improvement of people's living standard and education level, people realize the importance of health and their demands for medical conditions and services increase accordingly. The beam hardening artifacts and metal implant artifacts were reduced effectively by the HD energy spectrum CT scan, thus improving image quality. Besides, the incidence of hospital infection was reduced by accelerated rehabilitation nursing combined with PDCA circulation, thereby improving nursing satisfaction. However, there are still deficiencies. For example, the sample size is small, so it is necessary to expand the sample for the prospective study. In addition, the HD energy spectrum CT scan required large ray energy, which increased the radiation dose of patients. To sum up, the adoption of accelerated rehabilitation nursing combined with PDCA circulation quality based on the HD energy spectrum CT scan in the prevention and control of hospital infection in elderly patients who underwent elective orthopedic surgery was improved.

## Figures and Tables

**Table 1:** Total costs of stay (yuan).

Group	Treatment costs	Drug costs	Bed costs	Examination costs	Total costs
Observation group	408.45	78.93	167.34	136.72	791.44
Control group	1,544.67	1,362.56	875.42	286.31	4,068.96
*P*	<0.05	<0.05	<0.05	<0.05	<0.05

**Table 2:** Degree of nursing satisfaction (*n*, %).

Group	Very satisfied	Basically satisfied	Dissatisfied	Degree of satisfaction (%)
Observation group	28 (70)	9 (22.5)	3 (7.5)	92.5
Control group	16 (40)	15 (37.5)	9 (22.5)	77.5
*P*	<0.05	<0.05	<0.05	<0.05

## Data Availability

The data used to support the findings of this study are available from the corresponding author upon request.
